# The Dynamics of Addiction: Craving versus Self-Control

**DOI:** 10.1371/journal.pone.0158323

**Published:** 2016-06-28

**Authors:** Johan Grasman, Raoul P. P. P. Grasman, Han L. J. van der Maas

**Affiliations:** 1 Biometris, Wageningen University and Research Centre, Wageningen, The Netherlands; 2 Department of Psychology, University of Amsterdam, Amsterdam, The Netherlands; Oregon Health and Science University, UNITED STATES

## Abstract

This study deals with addictive acts that exhibit a stable pattern not intervening with the normal routine of daily life. Nevertheless, in the long term such behaviour may result in health damage. Alcohol consumption is an example of such addictive habit. The aim is to describe the process of addiction as a dynamical system in the way this is done in the natural and technological sciences. The dynamics of the addictive behaviour is described by a mathematical model consisting of two coupled difference equations. They determine the change in time of two state variables, craving and self-control. The model equations contain terms that represent external forces such as societal rules, peer influences and cues. The latter are formulated as events that are Poisson distributed in time. With the model it is shown how a person can get addicted when changing lifestyle. Although craving is the dominant variable in the process of addiction, the moment of getting dependent is clearly marked by a switch in a variable that fits the definition of addiction vulnerability in the literature. Furthermore, the way chance affects a therapeutic addiction intervention is analysed by carrying out a Monte Carlo simulation. Essential in the dynamical model is a nonlinear component which determines the configuration of the two stable states of the system: being dependent or not dependent. Under identical external conditions both may be stable (hysteresis). With the dynamical systems approach possible switches between the two states are explored (repeated relapses).

## Introduction

Although addiction refers to a very divers set of substances and behaviours, some common elements are generally recognized. In all addictions, after stopping, a strong desire arises to resume this frequently exerted action. When this is not possible, withdrawal symptoms will occur. This state of the mind, in which one is fully absorbed by the deprivation of some drug or activity, is called craving [1–3]. In this study craving is quantified by a variable *C* that ranges from 0 to 1. It arises as a result of dopamine activity, which in turn is switched on by other processes in the brain. In [4] craving from nicotine addiction has already been modelled by differential equations. Here, in a similar way, we study addiction, mainly in relation to alcohol consumption by using difference equations instead of differential equations. Differential equations as a tool for studying various aspects of the dynamics of addictive processes have attracted interest (e.g., [5–6]). Difference equations have been successfully used previously to describe biological systems in which quantities can only change at discrete points in a sequence of steps, for example the transmission and mutation of genes across generations of offspring [7], or the proliferation of a rabbit population by the famous Fibonacci equation. Difference equations in the context of alcohol abuse have also been considered, e.g., in [8]. One advantage of difference equations is that they are often more intuitively understood than differential equations, which makes it easier to communicate a model.

**Fig 1:**
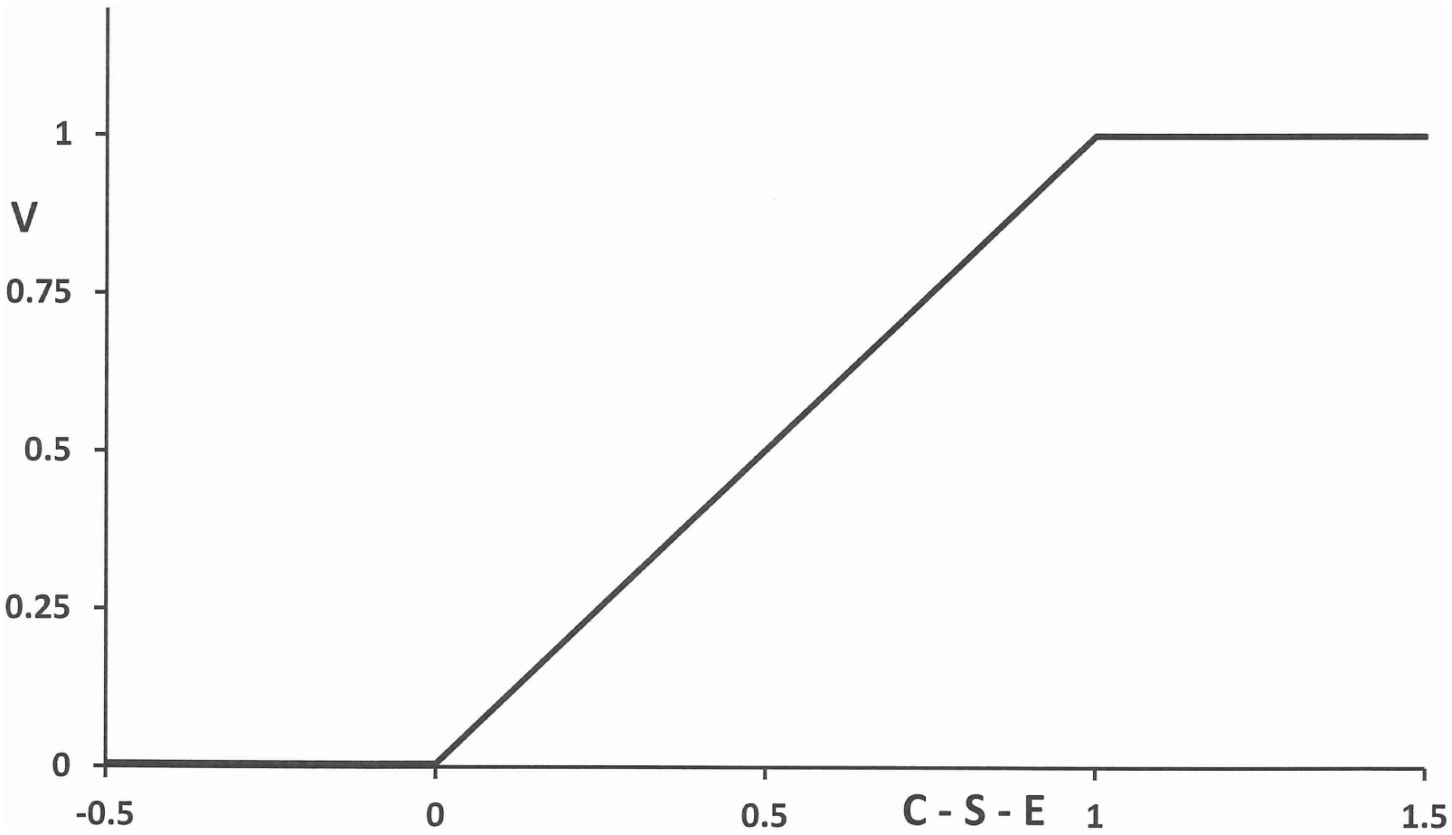
Dependence of addiction vulnerability *V* upon *C*, *S* and *E*.

A general factor in addiction is self-control. The effect *S* of self-control is introduced as a state variable. The other state variable *C* may undermine a person’s self-control. In addition to self-control, we include external control parameter *E*, referring, for instance, to the positive effect of societal measures. Peers may stimulate a person to continue or (re)start an addictive habit. This will lead to negative values of *E*. Addiction is quantified by the frequency of additive actions such as lighting a cigarette, drinking alcohol, or playing computer games. Its intensity (per unit of time) is represented by the variable *A*. It is influenced by the variables *C* and *S* and the parameter *E*. Assuming that these quantities have the same scale, we may write *A* = *A*(*C—S—E*). The argument *V* = *C—S—E* of this continuous function can be identified as addiction vulnerability [9] with *V* = 0 for *C—S—E* < 0 and *V* = 1 for *C—S—E* > 1. It is assumed that within the interval [0, 1] the quantity *V* is a linear function of *C—S—E* (Fig 1).

**Fig 2:**
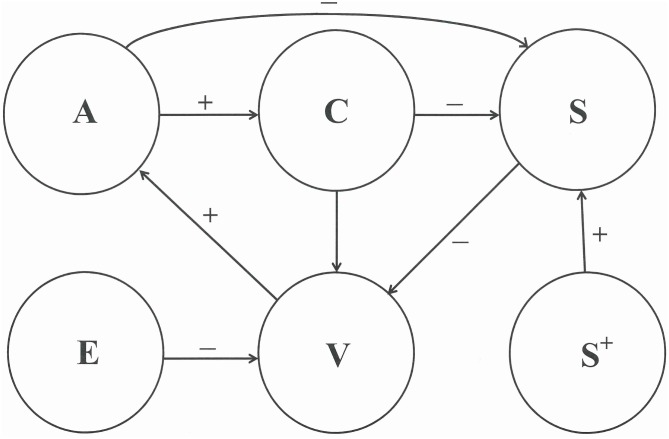
Vulnerability *V* is the sum of external forces *E*, self-control *S* and craving *C*, while acting out *A* is proportional to *V*.

Let the degree of self-control itself be quantified by a variable *U*. The variable *S*, the effect of self-control upon the suppression of addictive acts, is likely to be a monotonically increasing function of *U*. At some level *U* = *U*_0_ (> 0) there is no effect *S*(*U*_0_) = 0. The variable *U* only takes positive values, while *S* may be negative. The way the variables *A* and *C* may affect *S* can be incorporated directly in the model because of the one to one mapping *S* = *S*(*U*). It is understood [10] that self-control restores to a maximum level *U*^+^ after it has got depleted from encumbering experiences. At this maximum level the effect of self-control upon addictive behaviour equals *S*^*+*^ = *S*(*U*^*+*^). In Fig 2 a diagram is given that shows the way the variables and parameters, introduced above, affect each other.

This study deals with the dynamics of craving and self-control. In dynamical system theory one may employ either differential or difference equations for this purpose [11]. We choose here the latter, meaning that discrete time steps are taken which, in our case, have the size of one week. This may be justified by the fact that lifestyle exhibits a temporal cyclic pattern with a period of one week. For our model two functions must be defined that express the variables *C* and *S* at time *t* + 1 in values of *C* and *S* at time *t* [12]. Furthermore, variable *A* at time *t* depends on *C*, *S*, *E* at that time. Thus, there are three equations with *A*, *C* and *S* as variables. The quantities *E* and *S*^+^ are parameters and *V* is an auxiliary variable. In our dynamical system approach we have the possibility of including a stochastic input representing cues which may trigger an addiction [13–16].

If craving is different from zero (*C* > 0) one might say that a person is addicted. However, this study suggests a more complex dynamics. There may be two stable states to which the system is attracted: one with *C* = 0 and the other with *C* = *C* > 0. In some domain of the parameter space these states may even coexist. If the system is in the attraction domain of the one with *C* = 0, it does not require much effort to quit the potentially addictive activity; the person is not dependent of exerting the addictive activity [17]. In the other case the person is driven to the stable state of enduring addiction. It is convenient to label this trap as ‘being addicted’.

## Materials and Methods

The frequency of addictive acts *A* fluctuates at a time scale of hours with a pattern that also may depend on the day of the week. The type of addiction determines the variable *A*: for the intake of food and drugs the amount over one week is used. For addictive activities such as gambling [16], the frequency or duration over one week can be taken. We assume that *A* is a linear function of the addiction vulnerability *V*:

A=qV with V=min(1,max(0,C−S−E)), (1)

where *V* is the function as given in Fig 1 and *q* is the value of *A* that corresponds with an extremely high consumption level added over one week. It is assumed that the addictive acts take place at a time scale of hours. Being addicted means that one strives compulsively for an intensity at which one feels comfortable given the level of craving.

The dynamics of the process of addiction is described by a system of two coupled difference equations with the time *t* with a week as unit. We start with craving *C*(*t*). When a potentially addictive behaviour is prevented from being carried out, craving, being the desire for acting out the behaviour, dies off over time as it slowly becomes unlearned. Within the framework of difference equations, this can be mathematically expressed as

C(t+1)=(1−d) C(t),

where 0 < *d* < 1 is an unlearning parameter. Exerting the behaviour, however, has the contrary effect; acting out the behaviour will increase craving for engaging in it. The above equation is modified to include this effect

C(t+1)=(1−d) C(t)+γA(t),

where γ expresses the impact of *A* on *C*. However, as is often the case with stimulus intensities, the stronger the subjective intensity of a stimulus, the larger the change in stimulus has to be to further increase subjective intensity. In the same vein, we propose that the higher the sensation of craving *C*, the lower the impact of a one unit increase in *A* on this sensation. We therefore let the impact parameter γ decrease linearly as a function of *C*. In order to ensure that the impact is limited by a maximum, we take *γ* = *b*min(1, 1−*C*(*t*)), where *b* is the constant of proportionality of the impact of *A*, which we will coin the ‘cue sensitivity’ for reasons that will become apparent later on. The change in craving is therefore modelled by

C(t+1)=C(t)+bmin(1,  1−C(t))A(t)−d C(t), (2)

The decay of craving follows from (2) by setting *A* = 0; it is in the order of months, so we take *d* = 0.2.

A widely accepted tentative conceptualization of self-control, the ability to inhibit the acting out of the addictive behaviour, is that of a finite resource [10, 17–18]. Usage of this resource reduces availability and necessitates restauration to maximum capacity. In what follows, we will denote this maximum capacity by the parameter *S*^+^. Under normal circumstances restauration of the resource to maximum capacity occurs naturally. It is a process which finds its psychological foundation in the notion of psychological resilience [19]. In terms of the self-control, quantifying variable *S*, we may express this restauration process in a difference equation by

$$S(t+1)=S(t)+p\text{max}(0,\;S^{+}-\;S(t)),$$

which states that the level of the self-control resource at time *t* + 1 is whatever was left at time *t*, plus an amount that was restored. The amount restored is assumed to be proportional to the ‘left over capacity’: the body works harder to restore the resource the more it is depleted. The proportionality constant *p* can be thought of as a psychological resilience parameter. It is thought that in cases of addiction, self-control has to be constantly utilized at high levels which leads to complete depletion because restauration cannot keep up. This eventually undermines the capacity to exercise self-control. Various factors cause consumption of the self-control resource. It is well documented that most importantly in addiction, self-control is negatively impacted by both craving and the behaviour itself when it is acted out [20]. It is therefore natural to assume that the variable *S* decreases as craving *C* increases, or as the frequency of addictive acts, *A*, increases. Here we assume that both of these influences proportionally use up resource, thus, including the natural restauration process, we obtain the difference equation

$$S(t+1)=S(t)+p\text{max}(0,\;S^{+}-S(t))-h\;C(t)-kA(t).$$ (3)

**Fig 3:**
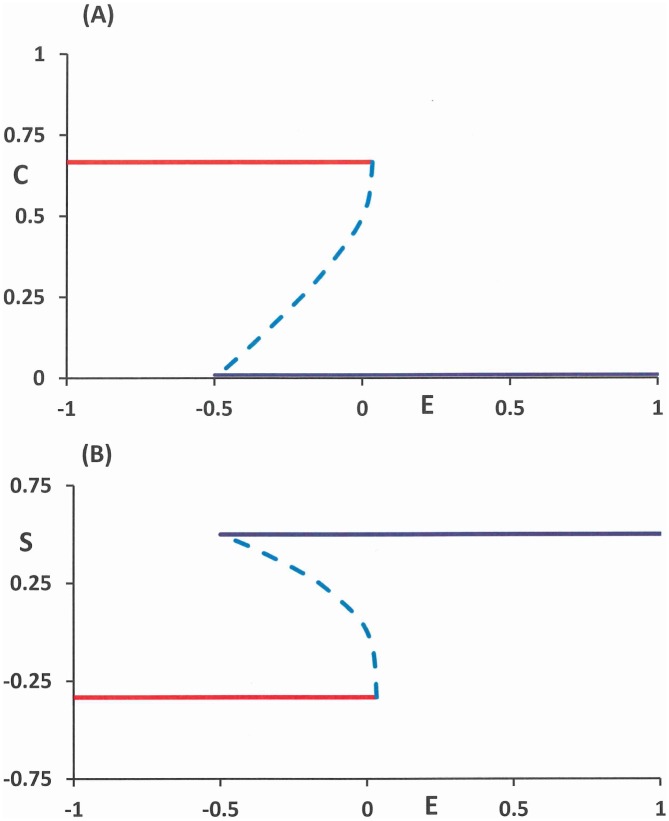
Equilibria of the system (1)–(3) for different values of the parameter *E*. There are two stable branches *C* = 0 and *C* = *C*(*E*) > 0 (solid) and one unstable branch (dashed). The branches are projected in the *E*,*C-*plane (A) and the *E*,*S-*plane (B).

The system of difference eqs (2) and (3) with constraint (1) constitute our general framework for studying the presence and absence of addictive states and possibilities for intervention. For alcohol addiction we take as a maximum of pure alcohol consumption over one week, *q* = 0.8 [kg], which equals 80 alcoholic beverages. The value of *b* can be derived by assuming that half the maximum consumption *A* = *q*/2 will result in half the maximum craving in the equilibrium state *C* = 1/2, it then follows that *b* = 2*d*/*q* = 0.5. The body must be able to restore self-control resource at a time scale that is smaller than that of craving fluctuations, or else self-control would very frequently be completely depleted even in healthy individuals: we take *p* = 2*d* = 0.4. Furthermore, we set the maximum capacity of self-control resource at *S*^+^ = 0.5. The last two terms of eq (3) are assumed to have the same strength: together they are of the same order as the restoring force: *h*/2 = *kq*/2 = *pS*^+^/2, so *h* = 0.2 and *k* = 0.25. Fig 3 shows the equilibria of eq (3) for *E* within the interval (-0.5, 0.3). There are two stable branches. A structure with two coexisting stable equilibria for a range of values of the parameter is also found in [21–22]. There the model consists of three state variables. This allows a slowly growing alcohol dependence followed by a rapid relapse.

## Results

When trying to apply the dynamical systems approach to the process of addiction in real life, it becomes clear that chance plays an important role. In particular cues [15] may lead to a change in behaviour that could not be foreseen. Cues are modelled as events that occur in time as processes having a Poisson distribution. We describe three types of episodes that may be experienced by persons who in their lifetime got involved in addictive activities: (a) getting addicted, (b) living with repeated relapses and (c) going into therapy.

### Cues and getting addicted

We consider the situation that an individual becomes a member of a community where customs exist that may lead to addiction: adolescents for example, at the brink of the legal drinking age, or adults who enter a new peer group or start a new living in a different culture. This may be mathematically expressed by the parameter *E* acquiring a sufficiently large positive value at the start, say *E* = 1, which then gradually decreases. In such a setting events may occur that trigger acting out addictive behaviours. These triggering events may be the presence of cues, such as social exposure or a mere opportunity of exerting addictive actions [13]. To include their influence, cues are modelled by modifying Eq 1 into

A(t)=qV(t)+f(R(λ(t));q), (4)

where *R* is a random nonnegative integer that corresponds to the number of cues encountered in a week. As cues come from many unrelated sources, it is reasonable to assume that *R*(λ(*t*)) constitutes a Poisson process with time dependent expected intensity λ. We will further assume that with each cue event the variable *A* increases with *q*/7 (one-seventh of the maximum consumption per week). It implies that *f*(*R*(*λ*(*t*));*q*) is not allowed to exceed the value *q*(1—*V*); otherwise the maximum *q* for one week would be exceeded. If that were the case, then consumption would be at its maximum, *A* = *q*. In the supporting information, S1 Program, an Excel program for carrying out the simulation, is given. The parameters *E* and λ become for the current case time dependent: *E* and λ, respectively, decrease and increase in time. The system can therefore be written as

C(t+1)=C(t)+bmin(1,  1−C(t))A(t)−d C(t), C(0)=0, (5a)

$$S(t+1)=S(t)+p\text{max}(0,\;S^{+}-S(t))-h\;C(t)-kA(t),\;S(0)=S^{+},$$ (5b)

E(t+1)=E(t)−dE, E(0)=1, (5c)

**Fig 4:**
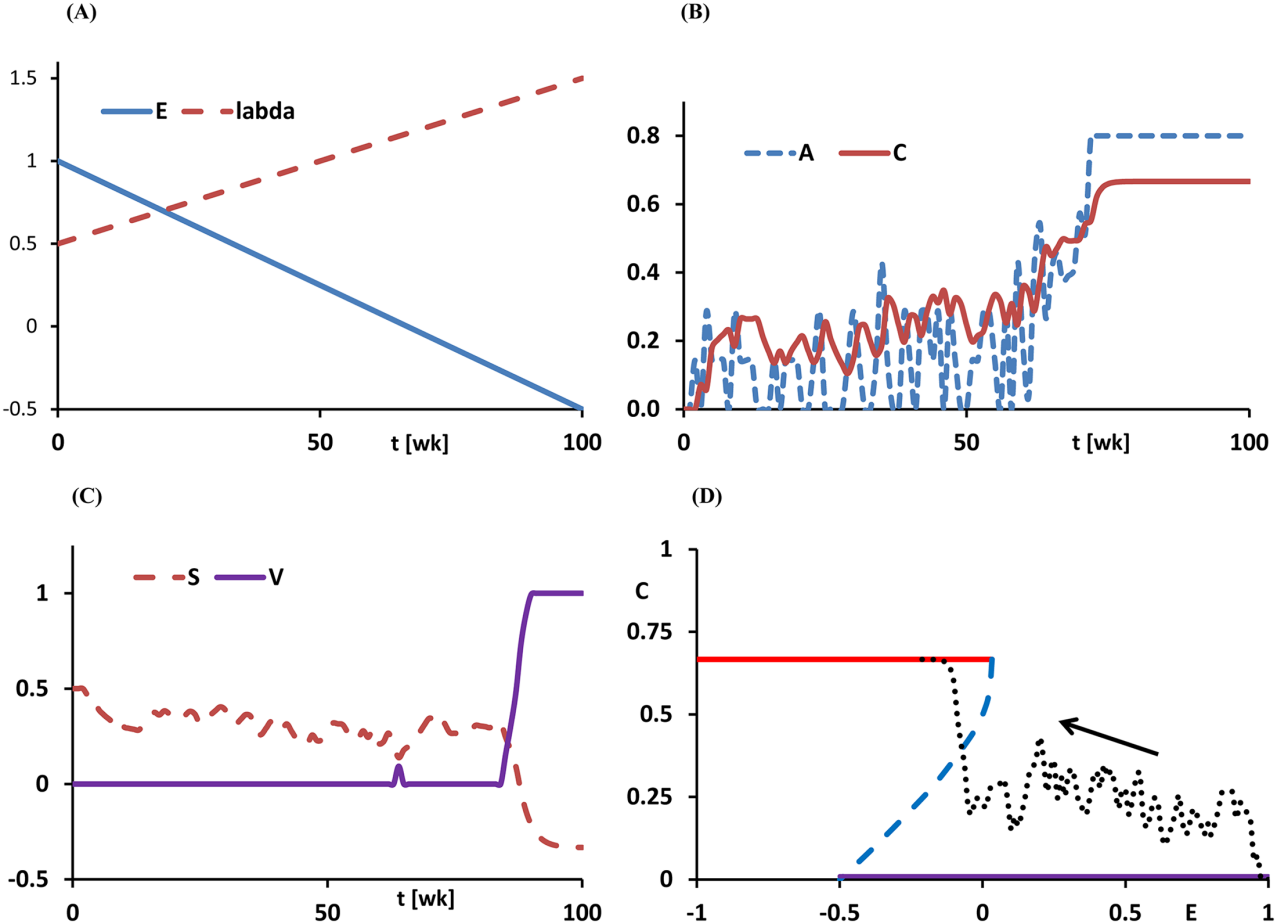
Getting addicted while changing lifestyle. Realisation of a solution of eqs (4) and (5): (A) Changing parameters *E* and λ. (B) Frequency of addictive acts *A* and the resulting craving *C*. (C) Decreasing self-control *S* and increasing vulnerability *V*. (D) Path projected in the *E*,*C*-plane.

λ(t+1)=λ(t)+dλ, λ(0)=0.5, (5d)

where *d*λ and *dE* denote the change of λ and *E* respectively. Eq 5a underlies our designation of the parameter *b* as reflecting ‘cue sensitivity’. To gain insight into the behaviour of these equations, we carry out a Monte Carlo simulation. In Fig 4 one realisation of a solution of the system (4) and (5) is depicted. Fig 4a shows the change in environmental influences *E* (new customs), and cue intensities λ. Fig 4b shows how alcohol consumption (the addictive behaviour *A*) fluctuates randomly in accordance with the random presence of cues, and covaries strongly with craving *C*. It is seen that when *E* decreases (as a function of time), *C* fluctuates at first considerably, but on average remains moderate. At a point were *E* is small enough however, *C* jumps to a very stable high level corresponding to a constant craving, and the system settles in the addicted state. Interestingly, it may be noted that from the level of craving *C* alone it cannot be concluded whether there is a switch to alcohol dependence at some time *t*. The addiction vulnerability *V* appears to be a better indicator for that purpose. A new lifestyle with external influences, bringing about activities that are potentially addictive, does not necessarily lead to a full addiction (*V* = 1). The stationary end values of *E* and λ determine the outcome.

### Repeated relapses

**Fig 5:**
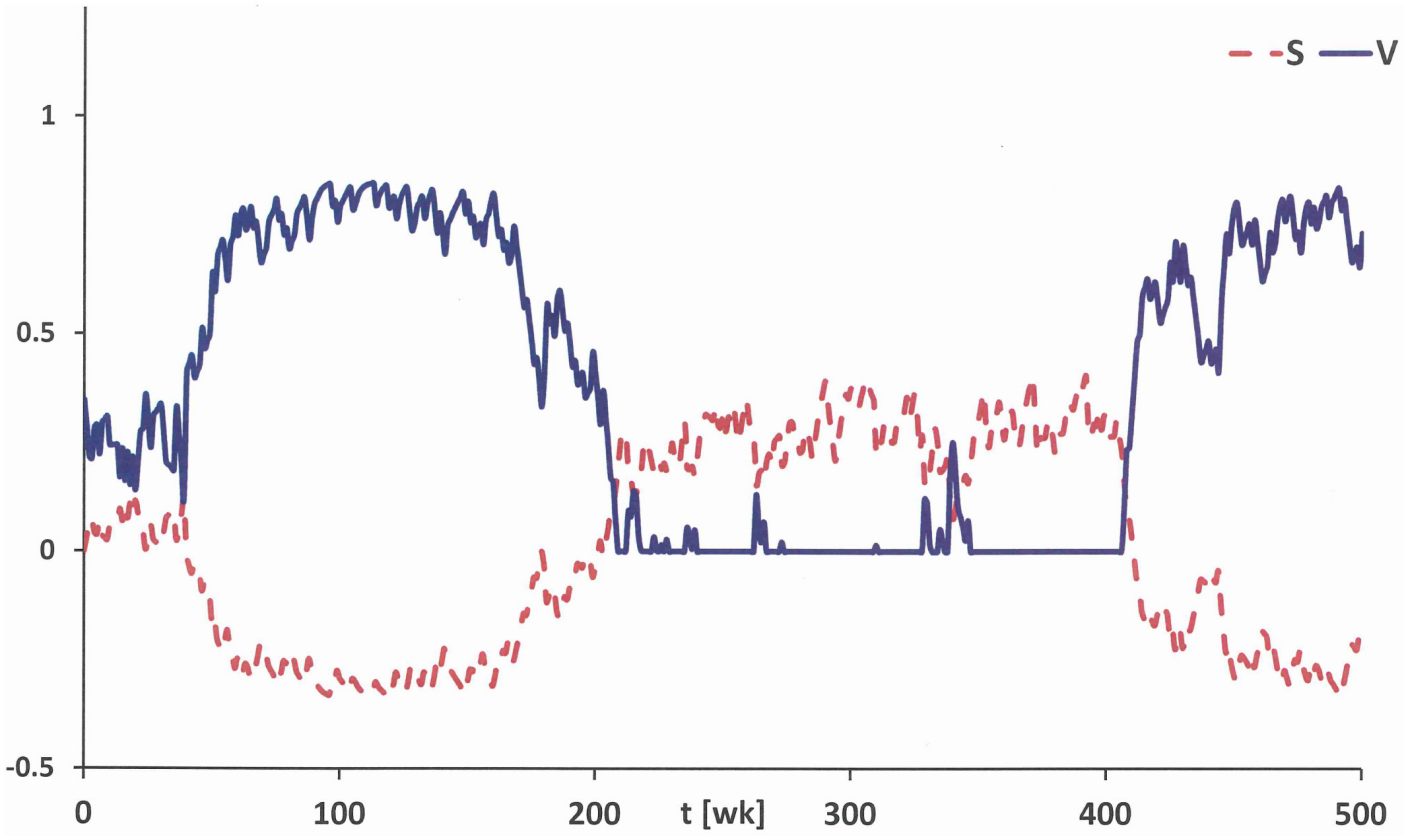
Simulation run of the stationary solution of eqs (4), (5a and 5b). Given is the effect of self-control *S* (dashed) and addiction vulnerability *V* (solid) for parameter values *E* = 0.1525 and λ = 1.

In a stationary environment with a fixed rate of Poisson distributed cues persons may exhibit a volatile addiction vulnerability. This may lead to repeated relapses triggered by a locally higher intensity of cues [23]. A time interval with locally a lower cue intensity may bring down the addiction vulnerability again. In Fig 5a realisation of the stationary solution of eqs (4), (5a and 5b) is given over a time interval of about 10 years. In [4] a similar temporal pattern for the dependence on nicotine has been found.

### Therapeutic intervention

The design of a recovery treatment should start with the definition of an acceptable future lifestyle of an individual after treatment. This lifestyle must correspond with a low addiction vulnerability and therefore requires acceptable values for the self-control parameter *S*^+^ and the cue parameter λ. Furthermore, the environmental effect *E* has to be taken into consideration. Let us set these parameters at values

$$S^{+}=0.5,\;\lambda =0.5,\text{ and }E=0.$$ (6)

The other parameters of Eqs (1)–(4) remain the same. With these parameter values, we should verify that the system has a stable low vulnerability state. Such a state exists indeed for λ = 0, in which the individual experiences zero craving (*C* = 0) and maintains high levels of self-control resource; see Fig 3. It should be noted that from this state, because of the stochastic cues (λ = 0.5), the stable high vulnerability solution can still be reached (again) in finite time [24]. However, the expected waiting time for such an event to take place is large and the expected return time will be short.

We consider the design of a therapeutic intervention that lasts for 7 weeks and study the chance of a success. During treatment the restoring force *pS*^+^ for the self-control *S* must be increased and the effect of cues should be decreased. The restoring force can be increased by either increasing the resilience parameter *p*, or the maximum self-control resource capacity *S*^+^, Here we concentrate on an intervention that accomplishes an increase in resilience, e.g., by way of restructuring cognitions in cognitive behaviour therapy. The cue induced effect can be reduced for instance by avoiding environments with high levels of cues associated with the addictive behaviour. It is assumed that during the treatment the self-control restoring force is doubled as a result of therapy, and that the possible effect of cues is halved:

p=0.8 and λ=0.25. (7)

With Monte Carlo simulation the effect of the treatment is analysed. Success/failure was measured by the value (larger/smaller than 0.5) of the vulnerability at 100 weeks after the start of the treatment. The success rate of 50 interventions is about 70%.

## Discussion

For the study of processes in various disciplines, the dynamical systems approach has turned out to be a valuable tool. We explored its applicability in the analysis of lifestyles with potentially addictive behaviour such as a high alcohol consumption. Various authors have presented dynamical systems models for different aspects of the addiction process, including linear systems of differential equations [6, 25], discrete time Markov chains [5], and nonlinear difference equations models [8]. A dynamical system describes the change in time of the state variables that constitute the system, which in this case are the degree of craving and the effect of self-control. Quantification of craving at a scale from 0 to 1 was followed by choosing a same scale for the other variable that influences addiction: the effect of self-control and external influences. Their joint effect upon addiction, put in the formula for the frequency of addictive acts (1), fits the definition of addiction vulnerability [9]. The dynamical eqs (2) and (3) are based on the assumption that change depends linearly upon the system variables with the exception of craving as it depends on addictive activity; it would otherwise result in a unbounded growth of the craving variable. This nonlinearity brings about that for certain parameter values there is more than one stable stationary solution. After the formulation of the model, parameters values had to be chosen. Some of the parameters depend on the type addictive action, while the ones, that are directly related to craving and the effect of self-control, were chosen such that effects of craving and self-control can compete. Our model does not allow a level of accuracy in the choice of parameters that one often finds in the physical sciences. Fortunately, the model turns out to be rather robust, meaning that a change in parameter values does not lead to a large change in the behaviour of the system.

The model that we present focuses on the nonlinear dynamics of several interacting psychological and behavioural variables that have been deemed important concepts in theories of addiction. The nonlinearity in the systems explains various phenomena in addiction such as the presence of discrete states in which the system persists unless it is critically triggered. For example, many people experience difficulties in moderating themselves in acting out an addictive behaviour. They either stay abstinent, or indulge. The abstinence state, although fixed, can become highly unstable—one episode of giving in may set the one off to turn to the other fixed state, the stable drinking state. Our model was inspired by nonlinear systems theory that typically display this type of behaviour. This idea to relate stable and unstable steady states in dynamical systems was previously proposed in [25]. Their model takes the perspective of a purely rational economic agent that maximises its future expected utility. Our model differs in that it takes into account variables that have found to be important psychologically, such as self-control and craving. Other approaches to the dynamics of addiction find their origin in behavioural economics [5], biochemistry and pharmacology at cellular level [26], and personality theory [6]. The behavioural economics model in [5] builds strongly on [25] and connects the model to various dopamine findings in rat studies and brain imaging, in which the unexpectedness of a reward plays a central role. Similar to our approach, the model in [6] focuses on the longer lifespan changes that addictive behaviour instils on personality. In particular, this model involves a system of linear differential equations focuses on changes towards the sensation seeking side of the extraversion axis. The model by [6] is also inspired by nonlinear dynamics, and employs a self-exciting autoregressive process model from the econometric literature on data in order to demonstrate characteristics of nonlinear dynamics in single subject time series.

By adding the effect of cues to the model in the form of a stochastic input, the dynamics of the system shows the uncertainty one also meets in daily life of persons who are at risk of getting addicted. The analysis of such a system requires many runs to assess the probability of possible outcomes given some initial state. We simulated the process when a person enters a new life stage with the risk of addiction. With the Monte Carlo method we also considered the case of an initial state with a person being addicted. It is directly followed by a phase in which self-control is stimulated and cues are brought down in a way comparable with a therapeutic intervention. The outcome of the treatment turned out to depend on chance: the intervention was effective in approximately 70% of the 50 cases. This chance is determined by the specific effectiveness of the intervention due to an increase of the psychological resilience and the decrease of the number of cues. Another factor that determines the intervention’s chance of success is self-control capacity of the individual, which was not addressed in the above simulations because it seems likely that this capacity can only be changed over much larger time scales (years). Individual differences in sensitivity to cue elicited craving have been related to major personality axes like the behavioural activation system (BAS), introversion and neuroticism [27]. How these personality traits relate to psychological resilience and self-control capacity remains to be seen. In the current model the relationship between a change in craving and exposure to cues is reflected in the ‘cue sensitivity’ coefficient *b*. The value of *b* was derived by assuming that the individual will be acting out at half maximum (half maximum alcohol consumption) at craving equilibrium state *C* = 1/2. For different individuals the parameter may differ in value, resulting in different acting out (alcohol consumption) levels at this equilibrium craving state. The model predicts that this relationship depends on vulnerability *V* through (4), and hence depend on environmental or social pressure. How these parameters relate to personality axes [27] remains to be taken in consideration.

## Supporting Information

S1 Program (XLSX)

## References

[1] Hill AJ. The psychology of food craving. P Nutr Soc. 2007;66: 277–285. 10.1017/S0029665107005502 17466108

[2] Ramirez J, Miranda R. Alcohol craving in adolescents: bridging the laboratory and natural environment. Psychopharmacology. 2014; 231: 1841–1851. 10.1007/s00213-013-3372-6 24363093 PMC4127892

[3] Caselli G, Spada MM. Desire thinking: What is it and what drives it? Addict Behav. 2015;44: 71–79. 10.1016/j.addbeh.2014.07.021 25128328

[4] Grasman J, Grasman RPPP, Van der Maas HLJ. Transitions in smoking behaviour and the design of cessation schemes. PLoS ONE. 2012;7(10): e47139 10.1371/journal.pone.0047139 23071738 PMC3469545

[5] Redish AD. Addiction as a computational process gone awry. Science. 2004;306: 1944–1947. 15591205 10.1126/science.1102384

[6] Caselles A, Micó JC, Amigó S. Cocaine addiction and personality: a mathematical model. Brit J Math Stat Psy. 2010;63: 449–480. 10.1348/000711009X470768 20030966

[7] Nowak MA, Boerlijst MC, Cooke J, Smith JM. Evolution of genetic redundancy. Nature. 1997;388: 167–171. 9217155 10.1038/40618

[8] Warren K, Hawkins RC, Sprott JC. Substance abuse as a dynamical disease: Evidence and clinical implications of nonlinearity in a time series of daily alcohol consumption. Addict Behav. 2003;28: 369–374. 12573687 10.1016/s0306-4603(01)00234-9

[9] Everitt BJ, Belin D, Economidou D, Pelloux Y, Dalley JW, Robbins TW. Neural mechanisms underlying the vulnerability to develop compulsive drug-seeking habits and addiction. Phil Trans R Soc B. 2008;363: 3125–3135. 10.1098/rstb.2008.0089 18640910 PMC2607322

[10] Hagger MS, Wood C, Stiff C, Chatzisarantis NLD. Ego depletion and the strength model of self-control: A meta-analysis. Psychol Bull. 2010;136: 495–525. 10.1037/a0019486 20565167

[11] Edelstein-Keshet L (1988) Mathematical Models in Biology. McGraw-Hill; 1988.

[12] Gelfand LA, Engelhart S. Dynamical systems theory in psychology: assistance for the lay reader is required. Front Psychol. 2012; 3, article 382. 10.3389/fpsyg.2012.00382 PMC3464058 23060844

[13] Moeller FG. Sex, stress and drugs cues in addiction. Am J Psychiat 169: 351–353. 10.1176/appi.ajp.2012.12010041 22476673

[14] Baschnagel JS. Using mobile eye-tracking to assess attention to smoking cues in a naturalized environment. Addict Behav. 2013;38: 2837–2840. 10.1016/j.addbeh.2013.08.005 24018227

[15] Snelleman M, Schoenmakers TM, Van de Mheen D. The relationship between perceived stress and cue sensitivity for alcohol. Addict Behav. 2014; 39: 1884–1889. 10.1016/j.addbeh.2014.07.024 25133978

[16] Stewart MJ, Yi S, Stewart SH. Effects of gambling-related cues on the activation of implicit and explicit gambling outcome expectancies in regular gamblers. J Gambling Studies. 2014;30: 653–668. 10.1007/s10899-013-9383-8 23588797

[17] Jones A, Christiansen P, Nederkoorn C, Houben K, Field M. Fluctuating disinhibition: implications for the understanding and treatment of alcohol and other substance use disorders. Front Psychiat. 2013;4, article 40. 10.3389/fpsyt.2013.00140 PMC3804868 24155728

[18] Inzlicht M, Schmeichel BJ. What Is ego depletion? Toward a mechanistic revision of the resource model of self-control. Persp Psychol Science. 2012;7: 450–463. 10.1177/1745691612454134 26168503

[19] Fletcher D, Sarkar M. Psychological resilience: A review and critique of definitions, concepts, and theory. Eur Psychol. 2013;18: 12–23.

[20] Muraven M, Collins RL, Nienhaus K. Self-control and alcohol restraint: an initial application of the self-control strength model. Psychol Addict Behav. 2012;16: 113–120. 10.1037//0893-164x.16.2.113 12079249

[21] Hufford MR, Witkiewietz K, Shields AL, Kodya S, Caruso JC. Relapse as a nonlinear dynamic system: application to patients with alcohol use disorders J Abnorm Psychol. 2003;112: 219–227. 12784831 10.1037/0021-843x.112.2.219

[22] Witkiewitz K, van der Maas HLJ, Hufford MR, Marlatt GA. Nonnormality and divergence in posttreatment alcohol use: re-examining the project MATCH data another way. J Abnorm Psychol. 2007; 116: 378–394. 17516769 10.1037/0021-843X.116.2.378 PMC2048690

[23] Cooney NL, Litt MD, Morse P, Bauer LO, Gaupp L. Alcohol cue reactivity, negative-mood reactivity, and relapse in treated alcoholic men. J Abnorm Psychol. 1997;106: 243–250. 9131844 10.1037//0021-843x.106.2.243

[24] Grasman J, Van Herwaarden OA. Asymptotic Methods for the Fokker-Planck Equation and the Exit Problem in Applications. Springer; 1999.

[25] Becker GS, Murphy KM. A theory of rational addiction. J Polit Econ. 1988;96: 675–700.

[26] Nicolaysen LC, Justice JB. Effects of cocaine on release and uptake of dopamine in vivo: Differentiation by mathematical modeling. Pharmacol Biochem Be., 1988;31: 327–335. 10.1016/0091-3057(88)90354-1 3072568

[27] Franken IHA. Behavioral approach sensitivity predicts alcohol craving. Pers Indiv Differ. 2002;32: 349–355.

